# Physics, chemistry and biology of functional nanostructures

**DOI:** 10.3762/bjnano.3.94

**Published:** 2012-12-11

**Authors:** Paul Ziemann, Thomas Schimmel

**Affiliations:** 1 Institute of Solid State Physics, Ulm University, D-89069 Ulm, Germany; 2 Institute of Nanotechnology (INT), Karlsruhe Institute of Technology (KIT), 76021 Karlsruhe, Germany

Nanoscience emerged as a field addressing phenomena that are strongly related to and influenced by their length scale being in the nanometer range. From the very beginning its interdisciplinary character became obvious. Meanwhile, after nanoscience had left its infancy, this characteristic feature developed even further by extension of the contributing disciplines: Initially it was supported and pushed forward by physics, materials science and chemistry, with a strong interrelation with semiconductor/information technology with their road maps for the downscaling of switching and storage devices. Today, biology and thus, in a natural way, medicine are included, opening new research areas within nanoscience. As a consequence of this development towards a real transdisciplinary field of science, worldwide collaborative research networks have been installed encompassing physics, materials science, chemistry and biology as well as various areas of engineering.

One such network with focus on “Functional Nanostructures” has been successfully active for almost ten years in Baden-Württemberg (Germany) [1]. Recent results from this network form the nucleus of the present Thematic Series *“Physics, chemistry and biology of functional nanostructures”* in the *Beilstein Journal of Nanotechnology*, complemented by contributions from international groups outside this network.

Besides the interdisciplinary approaches of nanoscience, its ongoing success can be attributed to at least three more necessary conditions:

- ***Significant progress in fabricating and controlling nanometer-sized objects and functional systems.*** Examples are synthesis and controlled positioning of various nanoparticles and macromolecules providing, finally, specific functions if arranged on suitable platforms in an optimized way. In the context of arranging nanoobjects, the exploitation of self-organization often in combination with suitable templates plays a major role. This aspect of the “bottom-up” methods opens routes towards novel nanolithographies, and due to the practical importance of the issue, the topic will be addressed in a number of contributions to the present Thematic Series.
- ***Continuous progress related to analytical tools and facilities*** allowing as complete as possible characterization of nanoobjects and devices. It is of special importance to gain information on the structure as well as the chemical composition and state of nanosytems in order to relate these to the various sought-after properties. With respect to tools, immediate examples are the continuous improvements of scanning-probe measurements such as scanning tunneling or scanning force microscopy (STM, AFM) [2] and their numerous variants often combined with scanning electron microscopy (SEM) or scanning helium ion microscopy (SHIM). Remarkable also are the breakthroughs in structural insight due to the advent of Cs-corrected high-resolution transmission electron microscopy (HRTEM) [3–4] or spectroscopic imaging in scanning TEM (STEM) [5]. Related to facilities, certainly the worldwide impressive progress of synchrotron facilities has made an important contribution, now providing beams with spot sizes even below 10 nm, thus promising the application of spectroscopies such as photoelectron emission microscopy (PEEM) or X-ray magnetic circular dichroism (XMCD) on a single nanoobject [6–7].
- ***Progress in the theoretical understanding of nanoscaled phenomena.*** We deem progress in theory necessary in order to gain feedback for testing new predicted phenomena or optimizing already existing nanosystems and devices.

All three of these conditions together with the given examples play a major role in the present Thematic Series, which, in turn, stands alongside previous series. Of special interest appear those reports dealing with self-assembly on solid surfaces, micro- and mesoporous solids, electrical transport through nanostructures, nanooptical aspects, organic–inorganic hybrids and properties of magnetic nanoparticles. A much broader view on biomimetic approaches can be found in [8]. The choice of these recommendations becomes obvious on summarizing the main topics of the present Thematic Series:

- Nanolithography approaches based on self-organized colloidal systems
- Experimental and theoretical description of electrical transport through nanostructures. Here, focus is put on the electrochemically controlled preparation of metallic point contacts [9–10]
- Magnetic behavior of nanoparticles and -wires
- Nanophotonics
- Effect of nanoporosity on the catalytic properties of Au oxidizing CO
- Theoretical description of organic building blocks such as polythiophenes. These molecules are especially interesting for organic solar cells [11] and have been analyzed also by STS [12].

Of course, the contributions to this Thematic Series form just a snapshot of the current activities focused on functional nanostructures. We hope, however, that our choice, which was guided by the idea to present interesting examples for the three general conditions given above, will be found inspiring by the readers as well. We give thanks to all colleagues for the valuable reports on their research and the Beilstein team for the engaged editorial work.

Paul Ziemann and Thomas Schimmel

Ulm, Karlsruhe, November 2012
